# Changes in primary care provider utilization by phase of care for women diagnosed with breast cancer: a CanIMPACT longitudinal cohort study

**DOI:** 10.1186/s12875-019-1052-2

**Published:** 2019-11-21

**Authors:** K. Decker, R. Moineddin, C. Kendell, R. Urquhart, N. Biswanger, P. Groome, M. L. McBride, M. Winget, M. Whitehead, E. Grunfeld, for the Canadian Team to Improve Community-Based Cancer Care Along the Continuum (CanIMPACT)

**Affiliations:** 1 CancerCare Manitoba, 675 McDermot Avenue, Winnipeg, Manitoba R0E 0V9 Canada; 2 University of Manitoba, 750 Bannatyne Avenue, Winnipeg, Manitoba R3E 0W2 Canada; 3 University of Toronto, 500 University Avenue, Toronto, Ontario M5G 1V7 Canada; 4 Dalhousie University, 1276 South Park Street, Halifax, Nova Scotia B3H 2Y9 Canada; 5 Nova Scotia Health Authority, 1276 South Park Street, Halifax, Nova Scotia B3H 2Y9 Canada; 6 Queen’s University, 62 Fifth Field Company Lane, Kingston, Ontario K7L 3N6 Canada; 7 BC Cancer Agency, 686 West Broadway, Suite 500, Vancouver, British Columbia V5Z 1G1 Canada; 8 Stanford University, 1265 Welch Road, Stanford, California, 94305 USA; 9 Ontario Institute for Cancer Research, 661 University Avenue, Suite 510, Toronto, Ontario M5G 0A3 Canada

**Keywords:** Primary health care, Breast neoplasms, Clinical decision-making

## Abstract

**Background:**

Primary care providers (PCPs) have always played an important role in cancer diagnosis. There is increasing awareness of the importance of their role during treatment and survivorship. We examined changes in PCP utilization from pre-diagnosis to survival for women diagnosed with breast cancer, factors associated with being a high user of primary care, and variation across four Canadian provinces.

**Methods:**

The cohorts included women 18+ years of age diagnosed with stage I-III invasive breast cancer in years 2007–2012 in British Columbia (BC), Manitoba (MB), Ontario (ON), and Nova Scotia (NS) who had surgery plus adjuvant chemotherapy and were alive 30+ months after diagnosis (*N* = 19,589). We compared the rate of PCP visits in each province across phases of care (pre-diagnosis, diagnosis, treatment, and survival years 1 to 4).

**Results:**

PCP use was greatest during treatment and decreased with each successive survival year in all provinces. The unadjusted difference in PCP use between treatment and pre-diagnosis was most pronounced in BC where PCP use was six times higher during treatment than pre-diagnosis. Factors associated with being a high user of primary care during treatment included comorbidity and being a high user of care pre-diagnosis in all provinces. These factors were also associated with being a higher user of care during diagnosis and survival.

**Conclusions:**

Contrary to the traditional view that PCPs focus primarily on cancer prevention and early detection, we found that PCPs are involved in the care of women diagnosed with breast cancer across all phases of care.

## Background

Breast cancer is the third most commonly diagnosed cancer in Canada and accounts for 13% of all cancers [1]. While the incidence of breast cancer has fluctuated, breast cancer mortality has decreased significantly in developed countries over the last 20 years [2]. In Canada, the breast cancer mortality rate decreased at least 44% since its peak in 1986 [1]. This has resulted in improved survival (Canadian five-year net survival between 84 and 88%), increased prevalence rates, and more women who need follow-up and survivorship care [1, 3, 4]. Primary care providers (PCPs) have always played an important role in the diagnosis phase of breast cancer care while oncologists and other specialists have managed patients’ care during treatment and survivorship [5]. However, there is increasing awareness of the critical role of PCPs during the treatment and survivorship phases of care particularly given increased breast cancer prevalence and survival. This expanded role is supported by studies that have found that PCPs provide care for treatment toxicities and comorbid conditions during treatment, and follow-up care that is as safe as oncologist care that includes not just breast cancer and late effects follow-up but care for comorbid conditions and ongoing preventive care. Moreover, women have reported being very satisfied with follow-up care from PCPs [6, 7]. To better understand the extent of PCP involvement in the care of breast cancer patients throughout the care continuum, we examined changes in the level of PCP utilization from pre-diagnosis to survival and inter-provincial variation across four Canadian provinces. We also examined factors associated with being a high user of primary care during pre-diagnosis, diagnosis, treatment, and survivorship.

## Methods

### Design

This study was conducted as part of a large, population-based retrospective cohort study (the Canadian Team to Improve Community-Based Cancer Care along the Continuum – CanIMPACT) that examined health care use for women diagnosed with breast cancer using linked administrative health data from British Columbia (BC), Manitoba (MB), Ontario (ON), and Nova Scotia (NS) [8]. Approvals were received from all relevant institutional research ethics boards (BC Cancer Agency/University of British Columbia Research Ethics Board, University of Manitoba Health Research Ethics Board, Health Sciences and Affiliated Hospitals Research Ethics Board at Queen’s University in Ontario, Nova Scotia Health Authority Research Ethics Board) as well as all relevant data access and privacy committees (BC Ministry of Health Chief Data Steward, MB Health’s Health Information Privacy Committee, ICES-Queen’s Privacy Office, NS Department of Health and Wellness Data Access Committee, Health Data Nova Scotia Data Access Committee). Due to provincial legislation and policy related to privacy issues, datasets were required to be analyzed separately within each province rather than being combined.

### Study population

We included women 18+ years of age who were diagnosed with stage I-III invasive breast cancer from 2007 to 2010 in BC or from 2007 to 2012 in MB, ON, and NS. The cohort was further restricted to women who had surgery plus adjuvant chemotherapy and were alive 30+ months after diagnosis in order to follow-up women through their treatment and early survivorship (*N* = 19,589). Women were excluded if they did not have curative surgery (i.e., lumpectomy or mastectomy), were diagnosed with a new primary cancer or metastases in the first year after the breast cancer diagnosis date, were not eligible for provincial health insurance from diagnosis to the end of follow-up, or had a breast cancer recurrence within 2 years of diagnosis. Recurrence was defined as a second course of chemotherapy (chemotherapy initiated more than 3 months after the end of the last chemotherapy) or chemotherapy that began 1 year plus 90 days after surgery. Patients were censored at time of evidence of recurrence, diagnosed with a new primary cancer, death, or the study end date. The maximum follow-up time was 7.5 years. We did not exclude women who were on chemotherapy 1 year after diagnosis as this is the recommendation for Herceptin.

The following time phases were examined: pre-diagnosis (six to 30 months before diagnosis), diagnosis (0 to 6 months before diagnosis), treatment (start of chemotherapy to 6 months post chemotherapy initiation), and survivorship (four 12-month intervals beginning 12 months after diagnosis).

### Variables and data sources

Detailed information about the CanIMPACT datasets and the source populations can be found in Groome et al. (2018) [9]. Briefly for this paper, provincial physician billing data were used to determine the number of PCP visits in each phase of care. Provincial cancer registries were used to identify the study cohort and provide information about date of diagnosis, stage, chemotherapy, postal code, and date of death. Provincial health insurance plan data provided demographic information (age, sex), date and type of surgery, and was used to determine comorbidity.

Age of diagnosis was separated into six groups (18–39, 40–49, 50–59, 60–69, 70–74, 75+). Comorbidity was determined using the Johns Hopkins Aggregate Diagnosis Groups (ADGs) (0–3, 4–5, 6–7, 8+) [10]. Area of residence was determined using postal code and classified as rural, rural-remote, rural unknown, rural-very remote, and urban [11]. Statistics Canada 2006 census data were used to estimate household income (categorized into quintiles from Q1, the lowest income quintile to Q5, the highest income quintile) based on their dissemination area (DA) of residence as a proxy measure for individual-level income. Number of years since immigration was determined using the Canadian Immigration and Citizenship database and was available only for ON and BC (non-immigrant, < 5 years, 5–10 years, > 10 years). In addition to number of visits as a measure of volume, a high user of primary care was defined based on the 90th percentile of the number of PCP visits at baseline weighted to the length of each phase.

### Statistical analysis

Analyses were conducted in each province using centrally-developed SAS macros to ensure a standardized approach. A Generalized Estimating Equations (GEE) approach with an autoregressive [1] covariance structure to account for repeated measures was used. Negative binomial regression was used to compare the rate of PCP visits across the phases of care. The risk of being a high user of primary care was modelled using logistic regression.

## Results

Table 1 shows the characteristics of the cohorts by province. The distribution of age and stage at diagnosis and income quintile were similar between provinces. A higher percentage of women in BC had low comorbidity (0 to 3, 37.1%) compared to other provinces (24–28%). A higher percentage of women in MB (16%) and NS (19.9%) lived in a rural-very remote location compared to BC (6%) or ON (2.3%). The total number of PCP visits (i.e., not the total number of women) from pre-diagnosis to survival 4 years after diagnosis was 28,441 in BC, 8163 in MB, 69,302 in ON, and 4664 in NS.

**Table 1:** Characteristics of the cohort by province

		British Columbia *N* = 4133 (%)	Manitoba *N* = 1472 (%)	Ontario *N* = 12,851 (%)	Nova Scotia *N* = 1133 (%)
Age group	18–39	276 (6.7)	130 (8.8)	1299 (10.1)	76 (6.7)
	40–49	1157 (28.0)	405 (27.5)	3302 (25.7)	330 (29.1)
	50–59	1412 (34.2)	514 (34.9)	4252 (33.1)	357 (31.5)
	60–69	1019 (24.7)	325 (22.1)	3059 (23.8)	263 (23.2)
	70–74	189 (4.6)	68 (4.6)	610 (4.7)	53 (4.7)
	75+	80 (1.9)	30 (2.0)	329 (2.6)	54 (4.8)
Comorbidity	0–3	1534 (37.1)	406 (27.6)	3584 (27.9)	272 (24.0)
	4–5	1092 (26.4)	346 (23.5)	3126 (24.3)	263 (23.2)
	6–7	746 (18.0)	317 (21.5)	2832 (22.0)	230 (20.3)
	8–9	472 (11.4)	204 (13.9)	1831 (14.2)	181 (16.0)
	10+	284 (6.9)	199 (13.5)	1478 (11.5)	187 (16.5)
Stage at diagnosis	I	1083 (26.2)	344 (23.4)	2881 (22.4)	333 (29.4)
	II	2182 (52.8)	775 (52.6)	7332 (57.1)	568 (50.1)
	III	868 (21.0)	353 (24.0)	2638 (20.5)	232 (20.5)
Income quintile	Q1 (lowest)	744 (18.0)	191 (13.0)	2031 (15.8)	179 (15.8)
	Q2	778 (18.8)	267 (18.1)	2393 (18.6)	218 (19.3)
	Q3	843 (20.4)	292 (19.8)	2536 (19.7)	235 (20.8)
	Q4	878 (21.2)	377 (25.6)	2839 (22.1)	263 (23.3)
	Q5(highest)	835 (20.2)	343 (23.3)	3011 (23.4)	236 (20.9)
Area of residence	Urban	3593 (86.9)	1071 (72.8)	11,249 (87.5)	795 (70.2)
	Rural	98 (2.4)	38 (2.6)	703 (5.5)	27 (2.4)
	Rural-remote	176 (4.3)	124 (8.4)	597 (4.6)	86 (7.6)
	Rural-very remote	248 (6.0)	235 (16.0)	300 (2.3)	225 (19.9)
Total number of primary care provider visits		28,441	8163	69,302	4664

Notes: Comorbidity was measured using Aggregated Diagnostic Groups; 0.1% were missing comorbidity in BC; 1.3% missing income quintile in BC, 0.1% in MB, 0.3% in ON, and < 0.6% in NS; 0.4% missing area of residence in BC, 0.1% in MB, < 0.03% in ON; 0% had a rural unknown residence in BC, ON, and BC; < 0.7% had a rural unknown residence in MB. Total number of primary care provider visits determined using physician billing data for all phases of care

Table 2 shows the provincial variation in PCP use by phase of care compared to pre-diagnosis levels. PCP use during diagnosis was higher in BC, MB, and ON but lower in NS compared to pre-diagnosis. PCP use was higher during treatment compared to pre-diagnosis in all provinces. However, the difference was most pronounced in BC where PCP use was over six times higher during treatment. In all provinces, there was a general trend that PCP use was greatest during treatment and subsequently decreased with each successive survival year. In BC, there was no difference in PCP use during survival year 1 compared to pre-diagnosis. PCP use during survival years 2, 3 and 4 in BC was significantly lower compared to pre-diagnosis. PCP use was higher during survival years 1 and 2 in MB compared to pre-diagnosis with no difference in use during survival years 3 and 4. In ON, PCP use was higher during survival years 1, 2, and 3 with no difference during survival year 4 compared to pre-diagnosis. PCP use was significantly higher in NS during survival year 1 and significantly lower during survival year 4 compared to pre-diagnosis.

**Table 2:** Variation in primary care provider utilization by phase of care and province (unadjusted)

		British Columbia	Manitoba	Ontario	Nova Scotia
		RR (95% CI)	RR (95% CI)	RR (95% CI)	RR (95% CI)
Phase of care	Pre-diagnosis	Ref	Ref	Ref	Ref
	Diagnosis	1.65 (1.61–1.70)	1.38 (1.32–1.43)	1.42 (1.40–1.45)	0.72 (0.61–0.84)
	Treatment	6.41 (6.21–6.63)	2.13 (2.02–2.25)	1.84 (1.80–1.88)	1.39 (1.32–1.46)
	Survival Year 1	1.02 (0.99–1.06)	1.24 (1.19–1.30)	1.19 (1.16–1.21)	1.28 (1.22–1.34)
	Survival Year 2	0.85 (0.82–0.88)	1.12 (1.07–1.17)	1.08 (1.05–1.10)	1.01 (0.93–1.10)
	Survival Year 3	0.59 (0.57–0.62)	1.05 (0.99–1.11)	1.03 (1.01–1.05)	0.93 (0.81–1.06)
	Survival Year 4	0.40 (0.38–0.43)	0.99 (0.93–1.06)	0.98 (0.96–1.01)	0.85 (0.75–0.97)

Notes: RR – Relative Risk; CI – Confidence interval

Table 3 shows the results of the analysis assessing the factors association with being a high user of primary care during treatment. The 90th percentile of the number of PCP visits at baseline was 15 visits for BC, 20 visits for NS, 19 visits for ON, and 20 visits for MB. Younger age was associated with lower odds of high PCP use in BC (age 40–49) and ON (age 18–59) relative to 60–69 year olds. In all provinces, a higher comorbidity score was associated with higher PCP use, and those with eight or more ADGs were consistently likely to be highest users of primary care. Income quintile was only associated with high PCP use in MB and ON. In MB, women who lived in areas with the second lowest income quintile (Q2) were more likely to have high PCP use than those living in areas with the highest quintile (Odds Ratio (OR) 1.82; 95% Confidence Interval (CI) 1.25–2.66). In ON, the likelihood of being a high PCP user decreased as income quintile increased. The likelihood of high PCP use increased with stage in BC and MB, while in ON, only those diagnosed with stage II cancers were more likely to have high PCP use compared to stage I. Women who lived in rural settings (i.e., rural, rural-remote, and rural–very remote) were more likely to be high PCP users compared to women who lived in urban areas in BC, MB, and ON. In NS, only women in rural-very remote areas were more likely to be high PCP users compared to urban areas (OR 1.52, 95% CI 1.05–2.20). Immigration was associated with high PCP use in BC but not in ON; women who had immigrated more than 10 years ago were less likely to be high PCP users compared to non-immigrants (OR 0.66, 95% CI 0.51–0.85). Finally, women who were high PCP users in the pre-diagnosis phase were more likely to be high users during treatment in all provinces.

**Table 3:** Factors associated with being a high user of primary care during treatment

		British Columbia (*N* = 4078)	Manitoba (*N* = 1467)	Ontario (*N* = 12,672)	Nova Scotia (*N* = 1131)
		OR (95% CI)	OR (95% CI)	OR (95% CI)	OR (95% CI)
Age group	18–39	0.83 (0.59–1.16)	0.98 (0.60–1.58)	0.70 (0.59–0.83)	1.30 (0.66–2.56)
	40–49	0.75 (0.61–0.94)	0.83 (0.59–1.17)	0.81 (0.72–0.91)	1.45 (0.94–2.25)
	50–59	0.89 (0.72–1.10)	1.01 (0.73–1.39)	0.88 (0.79–0.98)	1.39 (0.90–2.15)
	60–69	Ref	Ref	Ref	Ref
	70–74	1.05 (0.68–1.61)	1.55 (0.87–2.76)	1.13 (0.93–1.37)	1.64 (0.94–2.85)
	75+	1.57 (0.78–3.15)	1.20 (0.51–2.81)	1.28 (0.99–1.64)	
Comorbidity	0–3	Ref	Ref	Ref	Ref
	4–5	1.43 (1.18–1.72)	1.03 (0.73–1.46)	1.19 (1.05–1.34)	1.61 (0.94–2.75)
	6–7	1.44 (1.15–1.79)	1.24 (0.87–1.75)	1.47 (1.30–1.66)	2.26 (1.33–3.82)
	8+	2.63 (1.98–3.49)	1.59 (1.12–2.26)	2.03 (1.80–2.29)	3.93 (2.41–6.40)
Income quintile	Q1 (lowest)	0.97 (0.75–1.24)	1.36 (0.89–2.07)	1.34 (1.17–1.54)	1.37 (0.83–2.24)
	Q2	1.06 (0.83–1.36)	1.82 (1.25–2.66)	1.28 (1.12–1.46)	0.75 (0.45–1.26)
	Q3	1.18 (0.92–1.51)	1.18 (0.80–1.72)	1.25 (1.10–1.42)	1.21 (0.75–1.95)
	Q4	0.80 (0.63–1.00)	1.14 (0.79–1.62)	1.17 (1.03–1.33)	1.32 (0.83–2.08)
	Q5 (highest)	Ref	Ref	Ref	Ref
Stage	I	Ref	Ref	Ref	Ref
	II	1.20 (1.01–1.44)	1.43 (1.05–1.94)	1.12 (1.01–1.25)	1.07 (0.75–1.52)
	III	1.56 (1.24–1.97)	1.50 (1.05–2.15)	1.09 (0.96–1.24)	0.81 (0.52–1.28)
Area of residence	Rural	6.25 (2.52–15.51)	5.65 (2.82–11.32)	1.48 (1.24–1.77)	1.03 (0.61–1.76)
	Rural-remote	7.60 (3.54–16.33)	3.73 (2.49–5.58)	2.16 (1.81–2.58)	
	Rural-very remote	4.46 (2.65–7.48)	5.26 (3.83–7.21)	4.08 (3.20–5.22)	1.52 (1.05–2.20)
	Urban	Ref	Ref	Ref	Ref
Number of years since immigration	< 5 years	1.06 (0.70–1.61)	NA	0.98 (0.73–1.32)	NA
	5–10 years	0.76 (0.49–1.16)	NA	0.88 (0.66–1.16)	NA
	> 10 years	0.66 (0.51–0.85)	NA	0.92 (0.79–1.08)	NA
	Non-immigrant	Ref	NA	Ref	NA
Baseline high user	Yes	2.22 (1.47–3.37)	4.13 (2.63–6.48)	3.00 (2.63–3.43)	2.74 (1.73–4.34)
	No	Ref	Ref	Ref	Ref

Notes: Comorbidity was measured using Aggregated Diagnostic Groups; OR – Odds Ratio; CI – Confidence Interval

Results for the diagnosis phase (Additional file 1: Table S1) were similar to the treatment phase in that the odds of being a high primary care user increased as comorbidity increased in all provinces except NS. However, stage was only associated with high PCP use in MB, as was income quintile and area of residence in ON. Age was not associated with high PCP use during the diagnosis phase but immigration was in ON. Similarly, during the survivorship phases (Additional file 1: Tables S2 to S5), the odds of being a high primary care user increased as comorbidity increased (except BC and MB in year 4 and NS years 2, 3, and 4), and for high baseline use for all provinces and all years. A diagnosis of stage III breast cancer was associated with high PCP use in BC in survival years 1 and 2 and in MB during survival year 1. Immigration was only associated with PCP use in ON, although not for all years or subgroups. Age, income quintile, and area of residence were not consistently associated with high PCP use, but where associations existed, younger women were less likely to have high PCP use than women 60–69 and women who lived in lower income areas were more likely to have high PCP use than those who lived in the highest income areas.

## Discussion

We found that association of covariates with the likelihood of being a higher user of primary care varied between four Canadian provinces over the cancer care continuum for women diagnosed with stage I-III invasive breast cancer. The differences observed between provinces on the impact of different covariates on high PCP use may be related to differences in breast cancer screening program procedures and policies, differences in practice patterns during treatment, and differences in cancer agency and provincial recommendations regarding survivorship care. For example, differences observed between NS and other provinces in the association of various covariates with the likelihood of high PCP visits during the diagnosis phase of care may be related to the implementation of Nova Scotia Breast Screening patient navigators. Since 2000, a screening program navigator tracks and proactively facilitates further investigations for all women with imaging findings that require further testing, whether or not the women were screen detected or detected via a diagnostic mammogram. Because the navigator schedules all required imaging or biopsy appointments, women may be less likely to require a PCP visit. The navigator also provides women with informational and emotional support, which may also reduce a woman’s need to see a PCP. MB and ON have also implemented direct referral processes for women with suspected breast cancer. In MB, the screening program coordinates the first diagnostic test required after an abnormal screening mammogram and in ON, a breast assessment site arranges the surgical consultation appointment for women with a positive biopsy for about 50% of patients during the era of this study [12]. Other health care system factors that could impact the effect of covariates on the odds of high PCP use during diagnosis include differing wait times or access to specialists or other health care resources.

Our study also found that while breast cancer patients continued to visit their PCP during treatment in all provinces, there was a higher relative rate of PCP visits in BC. There may be various reasons for this observation including differences by province in how much PCPs are encouraged and supported to provide care for women during breast cancer treatment. Patient expectations and preferences may also be differ by province; generally, patients are increasingly consulting their PCP about treatment decisions [13, 14]. In a recent study that included 517 women diagnosed with early stage breast cancer, Wallner at al. found that up to one-third of PCPs reported involvement in multimodal breast cancer treatment decisions [15]. Moreover, older PCPs and those who more positively evaluated their ability to participate in cancer treatment decision making were more likely to participate [15].

Care during diagnosis involves investigative tests and procedures to establish the diagnosis [16]. Care during treatment involves monitoring and treatment of acute adverse physical and psychological impacts of cancer therapy, and management of comorbid conditions [16]. Care during survivorship is related to routine follow-up for recurrence and on-going surveillance for late adverse events [16]. Although PCP use increased during diagnosis and treatment, visits decreased with each survival year in all four provinces. By survival year 2, PCP visits in BC were significantly lower than during pre-diagnosis. This may be related to issues of awareness, responsibility, or feasibility in providing specific follow-up care by patients and/or PCPs. A qualitative study in ON found that some PCPs believed that survivorship care was not their responsibility while others suggested that PCPs were ideally suited to provide care because of their on-going relationship with patients [17]. The challenges to providing survivorship care by PCPs have been well documented and include a lack of time, increased workload burden, inconsistent educational preparation, limited knowledge, provider anxieties, and patient preference for oncology-led follow-up care [18–22]. Factors that may increase PCP participation in survivorship care include experience leading to higher confidence in managing follow-up for breast cancer patients, support and clear direction from oncologists about follow-up expectations and how to deal with potential problems, and engaging PCPs early in the patient’s cancer journey [5, 18, 23, 24]. Patient-specific survivorship care plans, which are endorsed by the American Society for Clinical Oncology and the Canadian Partnership Against Cancer, may also enhance the transition from specialist to PCP-provided care [25, 26].

The results of this study also highlight the factors that drive high primary care use in each phase of care and for each province. High comorbidity (8 or more ADGs) and baseline PCP use were important factors in all provinces for women who were alive at the start of the treatment phase of care. Therefore, women who had a greater need for care (e.g. due to comorbidity) prior to a diagnosis of breast cancer continued to have more PCP visits after diagnosis. Other important factors included advanced stage at diagnosis in BC and MB, rural residence in BC, MB, ON, and living in a lower income quintile in ON. A higher stage at diagnosis often requires more complex care with more physician visits while individuals living in rural areas may see their PCPs more often because PCPs are more accessible than oncologists who primarily provide care in urban areas. Interestingly, age was not consistently associated with high primary care use across the phases of care; this may be due to the strong correlation between age and comorbidities. Finally, women who were high baseline users of primary care were also more likely to be high users in each subsequent phase of care.

Strengths of this study include the use of population-based provincial cohorts that involved extensive work by the provinces to optimize data comparability. Despite this, we were unable to achieve optimum data analyses across provinces because of a lack of total data and file comparability [9, 27]. However, this study adds to the discussion of both the value and complexities of comparative analysis of health systems with the intention of encouraging appropriate data collection and linkage for similar health system-level studies in other jurisdictions [28].We did not examine potential barriers to primary care use. However, the Canada’s national health insurance program ensures that a lack of health insurance is not a barrier to care. Instead, morbidity is a major driver of high health care use [29]. Other potential barriers to care include not having a PCP (15.5% of Canadians reported that they did not have a PCP in 2017) [30], lower levels of income [31, 32], or issues related to health literacy [33].

## Conclusions

PCPs have traditionally been involved in the diagnosis phase of care; care during treatment and survivorship was seen as the responsibility of oncologists. However, we found that PCPs are involved in the care of women diagnosed with breast cancer throughout all phases of care and highest during treatment. Across the four provinces in this study, PCP use was higher during the diagnosis and treatment phases of care and early survivorship compared to pre-diagnosis and decreased in each successive survivorship year. In addition, women with comorbidities and those who were higher users of primary care prior to their cancer diagnosis were more likely to become high user of primary care during treatment. However, there were differences between the provinces; these variations in PCP use indicate potential practice and system-level opportunities to improve care for women diagnosed with breast cancer in Canada.

## Supplementary information

**Additional file 1: Table S1.** Factors associated with being a high user of primary care services during the diagnosis phase of care. **Table S2.** Factors associated with being a high user of primary care services during survival year 1. **Table S3.** Factors associated with being a high user of primary care services during survival year 2. **Table S4.** Factors associated with being a high user of primary care services during survival year 3. **Table S5.** Factors associated with being a high user of primary care services during survival year 4.

## Data Availability

The data that support the findings of this study are not publically available to ensure and maintain the privacy and confidentiality of individuals’ health information. Requests for may be made to the data stewards in each province (BC Ministry of Health Chief Data Steward, MB Health’s Health Information Privacy Committee, ICES-Queen’s Privacy Office, NS Department of Health and Wellness Data Access Committee, Health Data Nova Scotia Data Access Committee) but restrictions apply to the availability of these data, which were used under license for the current study, and so are not publicly available.

## Abbreviations

ADG: Aggregate Diagnosis Groups
BC: British Columbia
CI: Confidence Interval
DA: Dissemination Area
GEE: Generalized Estimating Eqs.
MB: Manitoba
NS: Nova Scotia
ON: Ontario
OR: Odds Ratio
PCP: Primary Care Provider
Q1: Quintile 1
Q2: Quintile 2
Q3: Quintile 3
Q4: Quintile 4
Q5: Quintile 5
RR: Risk Ratio
