# Supplementary material for: Changes in primary care provider utilization by phase of care for women diagnosed with breast cancer: a CanIMPACT longitudinal cohort study
Source: BMC Fam Pract. 2019 Nov 21;20:161. doi: 10.1186/s12875-019-1052-2 (PMC6873454; doi:10.1186/s12875-019-1052-2)
Supplement: Supplementary file 1 — Additional file 1: Table S1. Factors associated with being a high user of primary care services during the diagnosis phase of care. Table S2. Factors associated with being a high user of primary care services during survival year 1. Table S3. Factors associated with being a high user of primary care services during survival year 2. Table S4. Factors associated with being a high user of primary care services during survival year 3. Table S5. Factors associated with being a high user of primary care services during survival year 4. [file 12875_2019_1052_MOESM1_ESM.doc]

Supplemental data

Table S1. Factors associated with being a high user of primary care services during the diagnosis phase of care

|  |  | British Columbia (N=4078) | Manitoba (N=1467) | Ontario (N=12672) | Nova Scotia (N=1131) |
| --- | --- | --- | --- | --- | --- |
|  |  | OR (95% CI) | OR (95% CI) | OR (95% CI) | OR (95% CI) |
| Age group | 18-39 | 1.02 (0.71-1.48) | 1.20 (0.63-2.29) | 0.80 (0.64-1.00) | 0.57 (0.07-4.79) |
|  | 40-49 | 0.90 (0.70-1.14) | 1.30 (0.81-2.08) | 0.88 (0.76-1.03) | 0.63 (0.19-2.06) |
|  | 50-59 | 1.12 (0.90-1.39) | 1.04 (0.67-1.62) | 0.85 (0.74-0.99) | 0.59 (0.18-1.92) |
|  | 60-69 | Ref | Ref | Ref | Ref |
|  | 70-74 | 1.03 (0.69-1.56) | 0.86 (0.39-1.91) | 0.98 (0.76-1.26) | 0.54 (0.11-2.74) |
|  | 75+ | 1.47 (0.84-2.58) | 2.38 (0.97-5.83) | 0.96 (0.68-1.34) |  |
| Comorbidity | 0-3 | Ref | Ref | Ref | Ref |
|  | 4-5 | 1.82 (1.42-2.32) | 1.77 (0.91-3.42) | 2.32 (1.89-2.86) | 1.58 (0.26-9.64) |
|  | 6-7 | 2.62 (2.03-3.38) | 4.05 (2.23-7.36) | 3.59 (2.94-4.39) | 2.14 (0.38-11.98) |
|  | 8+ | 4.11 (3.17-5.33) | 6.42 (3.58-11.50) | 4.93 (4.05-5.99) | 2.69 (0.53-13.70) |
| Income quintile | Q1 (lowest) | 1.27 (0.97-1.68) | 1.32 (0.75-2.32) | 1.27 (1.06-1.52) | 1.31 (0.33-5.16) |
|  | Q2 | 1.20 (0.91-1.57) | 1.55 (0.93-2.57) | 1.28 (1.08-1.53) | 0.27 (0.03-2.43) |
|  | Q3 | 1.18 (0.90-1.54) | 0.99 (0.58-1.69) | 1.30 (1.10-1.55) | 0.88 (0.21-3.64) |
|  | Q4 | 1.12 (0.86-1.47) | 1.24 (0.77-2.00) | 1.18 (0.99-1.39) | 1.17 (0.32-4.29) |
|  | Q5 (highest) | Ref | Ref | Ref | Ref |
| Stage | I | Ref | Ref | Ref | Ref |
|  | II | 0.93 (0.77-1.14) | 1.54 (1.00-2.38) | 1.15 (1.00-1.32) | 1.04 (0.37-2.94) |
|  | III | 0.91 (0.71-1.17) | 1.84 (1.13-3.01) | 1.16 (0.98-1.38) | 1.00 (0.27-3.68) |
| Area of residence | Rural | 0.64 (0.33-1.25) | 2.09 (0.83-5.23) | 0.57 (0.42-0.78) | 0.53 (0.07-4.14) |
|  | Rural-remote | 1.07 (0.70-1.62) | 1.04 (0.59-1.84) | 0.55 (0.40-0.77) |  |
|  | Rural-unknown | NA | NA | NA | NA |
|  | Rural-very remote | 0.68 (0.45-1.02) | 1.01 (0.64-1.60) | 0.83 (0.54-1.27) | 1.60 (0.58-4.40) |
|  | Urban | Ref | Ref | Ref | Ref |
| Baseline high user | Yes | 4.24 (3.28-5.47) | 4.88 (3.14-7.57) | 7.31 (6.34-8.43) | 2.83 (0.90-8.83) |
|  | No | Ref | Ref | Ref | Ref |
| Number of years since immigration | <5 years | 1.57 (0.98-2.50) | NA | 2.38 (1.72-3.30) | NA |
|  | 5-10 years | 0.87 (0.49-1.55) | NA | 1.38 (0.99-1.91) | NA |
|  | >10 years | 1.27 (0.96-1.68) | NA | 1.59 (1.33-1.90) | NA |
|  | Non-immigrant | Ref | NA | Ref | NA |

Notes: Comorbidity was measured using Aggregated Diagnostic Groups.

Table S2. Factors associated with being a high user of primary care services during survival year 1

| Phase of care |  | British Columbia (N=2927) | Manitoba (N=1237) | Ontario (N=8773) | Nova Scotia (N=959) |
| --- | --- | --- | --- | --- | --- |
|  |  | OR (95% CI) | OR (95% CI) | OR (95% CI) | OR (95% CI) |
| Age group | 18-39 | 0.83 (0.50-1.38) | 0.91 (0.43-1.95) | 0.82 (0.60-1.13) | 0.53 (0.20-1.43) |
|  | 40-49 | 0.83 (0.61-1.15) | 1.32 (0.81-2.16) | 1.16 (0.94-1.43) | 0.87 (0.51-1.49) |
|  | 50-59 | 0.95 (0.71-1.28) | 1.07 (0.68-1.69) | 1.07 (0.88-1.31) | 1.00 (0.60-1.67) |
|  | 60-69 | Ref | Ref | Ref | Ref |
|  | 70-74 | 1.25 (0.72-2.16) | 1.66 (0.79-3.49) | 1.26 (0.89-1.76) | 1.41 (0.72-2.76) |
|  | 75+ | 1.58 (0.71-3.51) | 0.73 (0.22-2.42) | 1.03 (0.64-1.65) |  |
| Comorbidity | 0-3 | Ref | Ref | Ref | Ref |
|  | 4-5 | 1.85 (1.30-2.65) | 2.70 (1.39-5.24) | 1.73 (1.29-2.33) | 1.05 (0.53-2.08) |
|  | 6-7 | 2.82 (1.97-4.06) | 4.21 (2.22-7.99) | 3.26 (2.48-4.29) | 1.32 (0.67-2.59) |
|  | 8+ | 5.47 (3.82-7.82) | 6.06 (3.22-11.40) | 5.48 (4.21-7.13) | 2.81 (1.55-5.11) |
| Income quintile | Q1 (lowest) | 1.23 (0.85-1.79) | 1.20 (0.66-2.18) | 1.56 (1.23-1.98) | 0.88 (0.47-1.67) |
|  | Q2 | 1.33 (0.93-1.92) | 1.58 (0.93-2.68) | 1.27 (1.00-1.62) | 1.03 (0.56-1.89) |
|  | Q3 | 1.38 (0.96-1.98) | 1.05 (0.61-1.80) | 1.25 (0.99-1.57) | 0.87 (0.48-1.59) |
|  | Q4 | 1.14 (0.79-1.64) | 1.04 (0.63-1.72) | 1.29 (1.03-1.61) | 1.02 (0.58-1.80) |
|  | Q5 (highest) | Ref | Ref | Ref | Ref |
| Stage | I | Ref | Ref | Ref | Ref |
|  | II | 1.04 (0.79-1.37) | 1.48 (0.95-2.29) | 1.10 (0.92-1.32) | 1.42 (0.91-2.23) |
|  | III | 1.45 (1.04-2.01) | 1.93 (1.16-3.23) | 1.19 (0.94-1.49) | 1.24 (0.70-2.20) |
| Area of residence | Rural | 1.36 (0.64-2.90) | 1.01 (0.33-3.09) | 0.71 (0.48-1.04) | 1.47 (0.78-2.76) |
|  | Rural-remote | 1.58 (0.96-2.59) | 1.87 (1.08-3.23) | 0.58 (0.37-0.93) |  |
|  | Rural-unknown | NA | NA | NA | NA |
|  | Rural-very remote | 1.68 (1.06-2.67) | 1.19 (0.73-1.94) | 0.53 (0.27-1.04) | 1.45 (0.91-2.33) |
|  | Urban | Ref | Ref | Ref | Ref |
| Baseline high user | Yes | 3.24 (2.39-4.40) | 4.08 (2.55-6.52) | 5.63 (4.72-6.72) | 7.46 (4.37-12.71) |
|  | No | Ref | Ref | Ref | Ref |
| Number of years since immigration | <5 years | 1.20 (0.60-2.37) | NA | 1.27 (0.76-2.11) | NA |
|  | 5-10 years | 0.67 (0.28-1.64) | NA | 1.10 (0.69-1.73) | NA |
|  | >10 years | 1.29 (0.89-1.87) | NA | 1.45 (1.14-1.84) | NA |
|  | Non-immigrant | Ref | NA | Ref | NA |

Notes: Comorbidity was measured using Aggregated Diagnostic Groups.

Table S3. Factors associated with being a high user of primary care services during the survival year 2

| Phase of care |  | British Columbia (N=2801) | Manitoba (N=1208) | Ontario (N=8240) | Nova Scotia (N=522) |
| --- | --- | --- | --- | --- | --- |
|  |  | OR (95% CI) | OR (95% CI) | OR (95% CI) | OR (95% CI) |
| Age group | 18-39 | 0.74 (0.40-1.36) | 1.03 (0.47-2.23) | 0.64 (0.44-0.92) | 0.63 (0.13-3.15) |
|  | 40-49 | 0.85 (0.58-1.23) | 0.74 (0.42-1.29) | 0.74 (0.59-0.94) | 0.99 (0.43-2.28) |
|  | 50-59 | 1.19 (0.86-1.66) | 0.71 (0.43-1.18) | 0.93 (0.75-1.15) | 1.19 (0.53-2.66) |
|  | 60-69 | Ref | Ref | Ref | Ref |
|  | 70-74 | 1.68 (0.93-3.06) | 1.36 (0.61-3.02) | 1.35 (0.95-1.92) | 0.81 (0.25-2.59) |
|  | 75+ | 0.57 (0.16-2.02) | 0.76 (0.22-2.68) | 0.97 (0.57-1.63) |  |
| Comorbidity | 0-3 | Ref | Ref | Ref | Ref |
|  | 4-5 | 2.02 (1.32-3.09) | 1.28 (0.60-2.77) | 1.27 (0.91-1.77) | 10.81 (1.34-87.18) |
|  | 6-7 | 2.83 (1.84-4.35) | 2.17 (1.07-4.40) | 2.52 (1.88-3.39) | 5.86 (0.68-50.25) |
|  | 8+ | 5.14 (3.36-7.86) | 5.00 (2.59-9.66) | 4.28 (3.23-5.67) | 18.40 (2.40-141.34) |
| Income quintile | Q1 (lowest) | 1.34 (0.89-2.03) | 1.40 (0.70-2.82) | 1.70 (1.31-2.22) | 3.29 (1.14-9.45) |
|  | Q2 | 1.30 (0.86-1.95) | 2.91 (1.58-5.33) | 1.30 (0.99-1.70) | 2.20 (0.78-6.24) |
|  | Q3 | 1.03 (0.68-1.56) | 1.42 (0.75-2.71) | 1.52 (1.18-1.97) | 1.57 (0.53-4.66) |
|  | Q4 | 0.98 (0.65-1.48) | 1.40 (0.76-2.56) | 1.25 (0.96-1.62) | 2.03 (0.75-5.47) |
|  | Q5 (highest) | Ref | Ref | Ref | Ref |
| Stage | I | Ref | Ref | Ref | Ref |
|  | II | 1.15 (0.84-1.58) | 1.11 (0.69-1.80) | 1.21 (0.99-1.49) | 0.53 (0.26-1.05) |
|  | III | 1.54 (1.05-2.25) | 1.41 (0.79-2.50) | 1.28 (0.99-1.66) | 0.83 (0.36-1.94) |
| Area of residence | Rural | 1.78 (0.79-4.00) | 1.35 (0.38-4.83) | 0.71 (0.46-1.09) | 0.95 (0.35-2.55) |
|  | Rural-remote | 1.33 (0.75-2.36) | 1.46 (0.77-2.78) | 0.66 (0.41-1.08) |  |
|  | Rural-unknown | NA | NA | NA | NA |
|  | Rural-very remote | 0.87 (0.46-1.66) | 1.39 (0.81-2.39) | 0.46 (0.21-1.02) | 1.05 (0.45-2.44) |
|  | Urban | Ref | Ref | Ref | Ref |
| Baseline high user | Yes | 3.71 (2.66-5.19) | 3.48 (2.13-5.70) | 5.50 (4.53-6.67) | 3.53 (1.69-7.37) |
|  | No | Ref | Ref | Ref | Ref |
| Number of years since immigration | <5 years | 1.00 (0.43-2.30) | NA | 1.05 (0.57-1.93) | NA |
|  | 5-10 years | 2.02 (0.99-4.13) | NA | 2.05 (1.31-3.20) | NA |
|  | >10 years | 1.21 (0.80-1.84) | NA | 1.27 (0.96-1.67) | NA |
|  | Non-immigrant | Ref | NA | Ref | NA |

Notes: Comorbidity was measured using Aggregated Diagnostic Groups.

Table S4. Factors associated with being a high user of primary care services during survival year 3

| Phase of care |  | British Columbia (N=2028) | Manitoba (N=984) | Ontario (N=7866) | Nova Scotia (N=277) |
| --- | --- | --- | --- | --- | --- |
|  |  | OR (95% CI) | OR (95% CI) | OR (95% CI) | OR (95% CI) |
| Age group | 18-39 | 0.65 (0.32-1.33) | 0.78 (0.32-1.90) | 0.53 (0.35-0.82) | 0.33 (0.03-3.40) |
|  | 40-49 | 0.83 (0.54-1.28) | 0.47 (0.25-0.90) | 0.82 (0.64-1.06) | 0.89 (0.29-2.73) |
|  | 50-59 | 0.85 (0.56-1.28) | 0.47 (0.26-0.84) | 1.07 (0.86-1.34) | 0.20 (0.04-0.96) |
|  | 60-69 | Ref | Ref | Ref | Ref |
|  | 70-74 | 1.15 (0.55-2.41) | 1.35 (0.58-3.14) | 1.42 (0.98-2.07) | 0.69 (0.14-3.39) |
|  | 75+ | 1.43 (0.46-4.43) | 1.14 (0.31-4.13) | 1.24 (0.72-2.11) |  |
| Comorbidity | 0-3 | Ref | Ref | Ref | Ref |
|  | 4-5 | 1.98 (1.18-3.34) | 1.26 (0.54-2.96) | 1.58 (1.11-2.26) | 1.69 (0.28-10.28) |
|  | 6-7 | 2.79 (1.65-4.72) | 1.86 (0.83-4.20) | 3.05 (2.21-4.20) | 1.22 (0.18-8.16) |
|  | 8+ | 3.81 (2.22-6.55) | 2.99 (1.39-6.47) | 4.43 (3.24-6.05) | 2.41 (0.43-13.53) |
| Income quintile | Q1 (lowest) | 1.24 (0.75-2.04) | 2.58 (1.17-5.69) | 1.74 (1.31-2.31) | 6.92 (1.04-46.03) |
|  | Q2 | 1.08 (0.66-1.77) | 1.80 (0.83-3.90) | 1.38 (1.04-1.83) | 6.90 (1.12-42.56) |
|  | Q3 | 0.91 (0.55-1.51) | 1.28 (0.58-2.83) | 1.43 (1.09-1.88) | 3.91 (0.66-23.03) |
|  | Q4 | 0.91 (0.55-1.51) | 1.84 (0.90-3.75) | 1.36 (1.03-1.79) | 2.46 (0.38-15.80) |
|  | Q5 (highest) | Ref | Ref | Ref | Ref |
| Stage | I | Ref | Ref | Ref | Ref |
|  | II | 1.31 (0.89-1.92) | 1.47 (0.83-2.63) | 1.22 (0.98-1.50) | 1.84 (0.62-5.46) |
|  | III | 1.39 (0.87-2.24) | 1.06 (0.51-2.19) | 1.17 (0.88-1.54) | 0.90 (0.20-4.10) |
| Area of residence | Rural | 1.58 (0.58-4.35) | 0.57 (0.07-4.50) | 0.71 (0.45-1.12) | 0.53 (0.09-3.01) |
|  | Rural-remote | 1.80 (0.91-3.57) | 1.56 (0.74-3.27) | 0.28 (0.14-0.58) |  |
|  | Rural-unknown | NA | NA | NA | NA |
|  | Rural-very remote | 1.49 (0.74-2.97) | 0.79 (0.40-1.57) | 0.75 (0.38-1.47) | 1.69 (0.51-5.60) |
|  | Urban | Ref | Ref | Ref | Ref |
| Baseline high user | Yes | 5.02 (3.35-7.52) | 3.74 (2.09-6.68) | 4.97 (4.05-6.11) | 11.41 (3.19-40.91) |
|  | No | Ref | Ref | Ref | Ref |
| Number of years since immigration | <5 years | 1.12 (0.42-3.05) | NA | 1.23 (0.66-2.28) | NA |
|  | 5-10 years | 1.21 (0.45-3.29) | NA | 1.27 (0.75-2.14) | NA |
|  | >10 years | 1.49 (0.91-2.45) | NA | 1.24 (0.93-1.66) | NA |
|  | Non-immigrant | Ref | NA | Ref | NA |

Notes: Comorbidity was measured using Aggregated Diagnostic Groups.

Table S5. Factors associated with being a high user of primary care services during survival year 4

|  |  | British Columbia (N=1296) | Manitoba (N=716) | Ontario (N=6407) | Nova Scotia (N=167) |
| --- | --- | --- | --- | --- | --- |
|  |  | OR (95% CI) | OR (95% CI) | OR (95% CI) | OR (95% CI) |
| Age group | 18-39 | 0.94 (0.41-2.11) | 0.53 (0.18-1.54) | 0.56 (0.35-0.88) | 0.32 (0.04-2.49) |
|  | 40-49 | 0.74 (0.44-1.25) | 0.53 (0.27-1.07) | 0.76 (0.58-0.99) | 0.25 (0.06-1.13) |
|  | 50-59 | 0.96 (0.59-1.54) | 0.45 (0.23-0.88) | 0.80 (0.63-1.03) | 0.39 (0.09-1.69) |
|  | 60-69 | Ref | Ref | Ref | Ref |
|  | 70-74 | 1.28 (0.52-3.15) | 1.29 (0.46-3.64) | 1.35 (0.90-2.03) | 1.44 (0.15-13.54) |
|  | 75+ | 5.69 (1.95-16.54) | 2.71 (0.67-10.96) | 1.65 (0.94-2.92) |  |
| Comorbidity | 0-3 | Ref | Ref | Ref | Ref |
|  | 4-5 | 2.58 (1.49-4.49) | 2.04 (0.85-4.91) | 1.64 (1.11-2.43) | 1.59 (0.25-10.18) |
|  | 6-7 | 2.24 (1.22-4.13) | 2.22 (0.91-5.43) | 2.80 (1.95-4.02) | 0.80 (0.09-7.21) |
|  | 8+ | 5.69 (3.17-10.23) | 2.07 (0.84-5.10) | 4.48 (3.16-6.36) | 0.51 (0.05-4.77) |
| Income quintile | Q1 (lowest) | 2.00 (1.10-3.64) | 1.78 (0.77-4.10) | 1.48 (1.09-2.01) | 0.45 (0.03-6.36) |
|  | Q2 | 1.51 (0.84-2.70) | 1.04 (0.43-2.51) | 1.17 (0.86-1.60) | 2.43 (0.42-13.92) |
|  | Q3 | 1.45 (0.80-2.63) | 1.21 (0.54-2.72) | 1.20 (0.88-1.63) | 1.24 (0.21-7.35) |
|  | Q4 | 1.04 (0.57-1.92) | 1.44 (0.68-3.05) | 1.04 (0.76-1.40) | 1.94 (0.35-10.77) |
|  | Q5 (highest) | Ref | Ref | Ref | Ref |
| Stage | I | Ref | Ref | Ref | Ref |
|  | II | 0.97 (0.64-1.48) | 0.86 (0.48-1.56) | 1.20 (0.95-1.52) | 0.77 (0.20-2.90) |
|  | III | 1.18 (0.69-2.02) | 0.65 (0.29-1.43) | 1.10 (0.80-1.51) | 0.66 (0.13-3.44) |
| Area of residence | Rural | 0.67 (0.14-3.19) | 0.66 (0.08-5.32) | 0.63 (0.36-1.09) | 0.51 (0.07-3.75) |
|  | Rural-remote | 1.53 (0.70-3.34) | 0.96 (0.38-2.45) | 0.66 (0.38-1.16) |  |
|  | Rural-unknown | NA | NA | NA | NA |
|  | Rural-very remote | 1.09 (0.46-2.59) | 1.03 (0.51-2.09) | 0.25 (0.08-0.83) | 0.62 (0.14-2.73) |
|  | Urban | Ref | Ref | Ref | Ref |
| Baseline high user | Yes | 3.51 (2.13-5.79) | 4.30 (2.11-8.73) | 4.95 (3.94-6.22) | 34.51 (5.02-236.97) |
|  | No | Ref | Ref | Ref |  |
| Number of years since immigration | <5 years | 0.85 (0.27-2.67) | NA | 1.97 (1.08-3.60) | NA |
|  | 5-10 years | 0.47 (0.10-2.18) | NA | 1.26 (0.69-2.29) | NA |
|  | >10 years | 1.10 (0.60-2.03) | NA | 1.00 (0.71-1.42) | NA |
|  | Non-immigrant | Ref | NA | Ref | NA |

Notes: Comorbidity was measured using Aggregated Diagnostic Groups.
